# Webconference mixed with virtual slides as a pedagogical tool to improve pathology practice in the French Midi-Pyrenees area

**DOI:** 10.1186/1746-1596-8-S1-S44

**Published:** 2013-09-30

**Authors:** ML Ranty, C Guilbeau-Frugier, M Jacob, P Gil, E Uro-Coste, Y Nicaise, MB Delisle

**Affiliations:** 1 Service d'Anatomie Pathologique et d’Histologie-Cytologie, Hôpital de Rangueil, 1 avenue Jean Poulhès TSA 50032 31403 Toulouse, France; 2 Toulouse Paul Sabatier University, DTSI, 118 Route de Narbonne 31062 Toulouse cedex 09, France

## Background

The pathology is a discipline located between basic science and clinical practice. Tissue and / or cellular injury could be seen as a triptych with in upstream pathophysiological mechanism and in downstream the resulting symptoms.

In recent years, pathology, an essential step of clinical diagnosis, is being more complex integrating complementary technics (e.g. molecular biology), creating new classifications or new recommendations. So pathologists’specialization in one field is becoming a necessity. Unfortunately, since 1999, the French pathologists growth rate is negative and demographic previsions estimates that in 2050 pathologists’ population should decrease by 50%. This crisis in vocations obliged pathologists to provide continuing education to their peers, share experiences but also to reform the teaching of the discipline during medical studies to make it more accessible and attractive. The development of digital imaging in this both areas has been a greatly helpful.

In France, a group of pathologists established in 1977 under the name ADICAP (“association for the development of informatics in pathology”) helped the creation of adequate informatics tools [1]. Worldwide, the pathologists were among the first to use tools of telemedicine, especially in countries with difficult geographical conditions such as Norway or Canada. However, for 25 years, the difficulties of digital transmission and the static and selective state (selection bias in the sent fields) of numeric photography had left this technology underutilized [2]. In the 2000s, development of the virtual slides technology (virtual slides scanner) and of telepathology, allowed diversification and wider use of digital imaging in pathology. It had been now widely used in Canada to exchange views particularly in cases of diagnostic emergency as extemporaneous examinations [1]. In 2010, physicians in three Northern Ontario communities had been virtually linked at all times to pathology specialists allowing frozen section examination on line [3]. It had been also used for teaching and universities, particularly in the U.S. and Switzerland, where virtual imaging have replaced conventional microscope. In France these technologies are progressively taking place both in the field of education and in the exchange of expertise. In Toulouse-Rangueil hospital, the pathology department has chosen to focus on education as the training provided to senior pathologists and to medical students.

## Methods

Slides were scanned on « NDP Nanozoomer » Hamamatsu, in Rangueil University Hospital, in Toulouse. The virtual slides were loaded in to a computer server, in Toulouse-Paul Sabatier University. Hamamatsu provided the program patch with the NDP viewer, as a pilot program.

### For continuing education

The Paul Sabatier University (TICE department, Direction of Technology and Information Systems) provided to the university community a web conferencing platform Adobe Connect Pro. This platform, web-based, used Flash technology present on 98% of computers. It offered not only a videoconferencing service but also a collaborative workspace (document sharing, screen or application sharing, instant messaging, whiteboard...) organized in modules. This type of system offered flexibility and brought a wealth of services in a nomadic environment. All medical data are anonymous.

**Figure 1:**
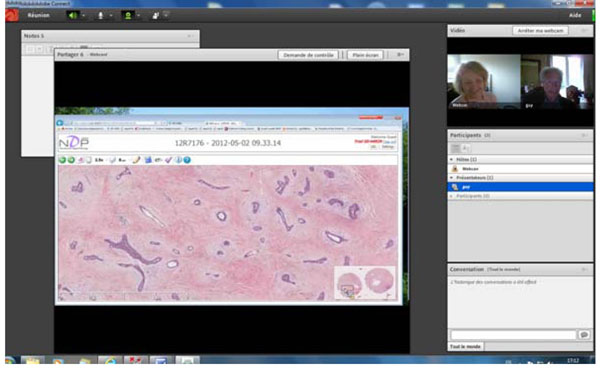
Virtual class visible by the senior pathologist in training.

Virtual meetings between pathologists: To access the platform, only an internet-connected computer, a webcam and a microphone was needed (Figure 1). The access was secured by a code.

### For theoretical teaching

We integrated the virtual slide in presencial sessions in small numbers (30 students per group, promoting divided into 5 groups, 10 sessions). Each student had a microscope and glass slides with a representative lesion. Each slide was previously scanned. The virtual slide was projected and commented on by a teacher. The student had to the area of interest selected by the teacher at low magnification and then at higher magnification leading to the diagnosis. Questionnaire of satisfaction was distributed to students and teachers at the end of the first teaching session and at the end of the sessions cycle (10th session). To enable students to visualize the virtual slide out of teaching session, a web-site is in process. It would allow the student to access to digital slides but also to recapitulative picture, with a brief explanation and a summary of what to remember. Normal tissue slides would also be available. Student would access the site through a custom code. It is not yet planned to grant access beyond the teaching year or to allow students external of our University to access the site. Thinking is still in progress on these points.

## Results of questionnaire of satisfaction

### For continuing education

This virtual organization had been ready for three months. To date, three meetings were organized. Members are satisfied with the ease of use and the gain of time. Some technical details are set up according to the settings of the individual participants, such as setting the webcam. Some participants, less in touch with computers, were a bit stressed and technical assistance was required.

### For theoretical teaching

**Figure 2:**
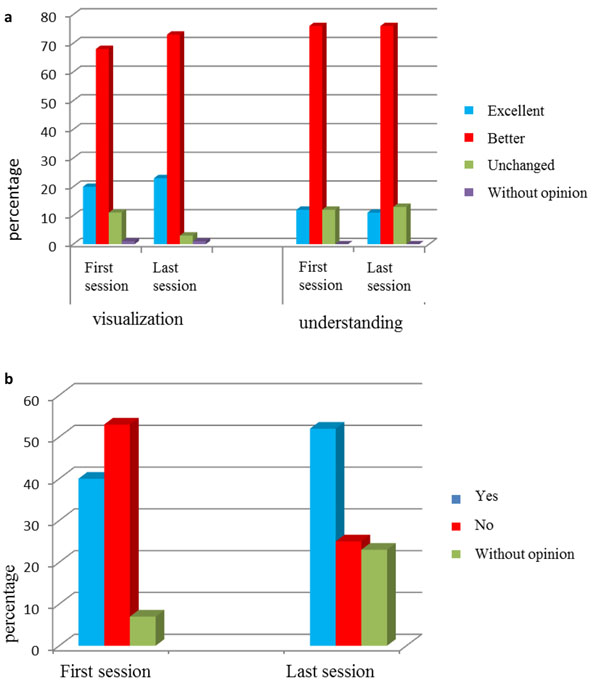
a) Students opinion on virtual slides for visualization and understanding at the first and last session. b) students opinion replacement of the microscope by the virtual slide at the first and last session.

No technical problem occurred during teaching session. All students answered to the satisfaction query. Students, at the end of the first session, found that visualization (ability in detecting and observing the interest area) and understanding was better (Figure 2a). At the end of 10 sessions, opinion upon the visualization of lesions had improved since 23% of students have found it excellent and 73% better. Understanding remained unchanged.

Teachers had same opinion (visualization enhanced 78% or excellent 22% and understanding better 78% or excellent 12%). The two less experienced teachers felt stressed by changing their teaching habits. It was interesting to note that students had not noticed this stress (95% of students found them perfectly relaxed).

At the end of the first session the majority of students (53%) would not give up the microscope. Nevertheless at the end of 10th session, they were only 25% (52% favorable to give up the microscope and 23% without opinion). All teachers though that virtual slides would be more beneficial than microscope. At the end of 10th over, 77% of students recommended to go on teaching with virtual slides however, 7% thought that it should be better to give up and 15% had no opinion (Figure 2b).

## Discussion

Virtual microscopy is undeniable progress that has many applications in education.

This technology allowed organization of virtual group learning events for continuing education sound and images were travelling by web [4]. The virtual slides could be observed by everybody, to the extent that it was authorized, with the possibility for every one to speak, to remote on the virtual slide to show a particular area. A moderator gave the right to speak or to move on the slide. Everyone could see the same area of the slide at the same time. It was possible to take memos concomitantly in a specific area and to save them on the computer or send them by mail. Participants can view the slides prior to the meeting, provided they are received in sufficient time to allow the digitization and online procedure. In this context of penury of pathologists but also in an economic standpoint, this virtual meeting, provides continuing education for seniors, lifting the geographical barrier, thus saving travel time. It improves practices. It allowed too the construction of a virtual slide box, ensuring deepening and maintenance of the latest knowledge. So, it allows the participation of people discouraged by the duration of transport. In our department, requests for such meeting are increasing.

In theorical education, as in our data, virtual slides have been regarded favorably by students improving visualization and understanding [5-7]. It eliminates the three most frequent complaints: the focus, the binocular vision difficulty and the identification of the area of interest (virtual slides can be annotated). In universities which have introduced this technique for many years, particularly in the U.S., teachers objectified better success in practical exams [8]. Most studies have observed that students preferred the virtual slides to the microscope [5-7]. The computer is perfectly mastered by the young generations, integrated into their world and do not necessitate special competence. In our study, teachers believed that the technique would be acclaimed, students needed to be convinced. They think that knowing how to use a microscope belongs to their formation. This skepticism was found in other studies in the literature, where it could take 2-3 years before a massive acceptance (95%), even if the technic was gradually introduced [8-10]. Digital slides require no particular training for teachers. In our study, teachers were experienced and inexperienced, someone had limited computer competency. No technical problem occurred. It is interesting to note that most experienced teachers were also more motivated and more enthusiastic. Young teachers have considered, at the first session, the virtual slide as additional stress before they feel it as comfortable. Our learning website is still under development and no evaluation is yet available.

## Conclusion

Virtual slides in pathology teaching were particularly welcomed by students as by teachers. Highly praised, the virtual slide, or virtual microscope, improves understanding and visualization and has educational application. It is powerful for senior pathologist continuing education e-learning in association with webconference. The virtual slides can be viewed on any computer, via a website, provide support for an examination or creation of a database of images ...

## Competing interests

The authors declare that they have no competing interests.

## Authors’ contributions

M-L R: corresponding author, in charge of continuing education

CG-F: in charge of theoretical education for medical students. Contribution of this author is equally to corresponding author.

MJ: technical assistance and creation of web-site.

PG: gestion of server.

EU-C: participation of elaboration of virtual environment and pedagogical support.

YN: technical support and scanning slides.

M-DB: participation of elaboration of virtual environment and pedagogical support.
